# Supplementary material for: An Innovative Approach to Alleviate Zinc Oxide Nanoparticle Stress on Wheat through Nanobubble Irrigation
Source: Int J Mol Sci. 2024 Feb 5;25(3):1896. doi: 10.3390/ijms25031896 (PMC10855730; doi:10.3390/ijms25031896)
Supplement: Supplementary file 1 [file ijms-25-01896-s001.zip › ijms-2851286-supplementary.pdf]

Supplementary Materials

Table S1. Effect of nanobubble watering with zinc oxide nanoparticles on activities of C-, N- and P-degrading enzymes in soil ($n = 3$).

		BG Activity (nmol g^{-1} h^{-1})			LAP Activity (nmol g^{-1} h^{-1})			NAG Activity (nmol g^{-1} h^{-1})			NP Activity (nmol g^{-1} h^{-1})		
		**mean**	**SE**	**sig**	**mean**	**SE**	**sig**	**mean**	**SE**	**sig**	**mean**	**SE**	**sig**
ZnO0	W	29.19	0.53	b	69.763	3.907	b	197.661	6.570	b	135.886	4.781	b
	nW	29.03	0.69	b	45.996	5.776	c	198.832	3.637	b	165.023	1.742	a
ZnO500	W	26.05	0.24	c	111.868	4.294	a	226.355	13.597	ab	163.851	1.130	a
	nW	35.59	0.21	a	80.146	9.785	b	258.666	16.400	a	160.261	7.539	a
ZnO			**			**			**			*	
nW			**			n.s.			n.s.			*	
ZnO × nW			**			n.s.			n.s.			**	

ZnO_{500} and ZnO_0 indicate irrigation with/without 500 mg L^{-1} ZnO solution, while W and nW indicate normal water and nanobubble-containing water. n.s., no significant difference; ** $p < 0.01$; * $p < 0.05$. Different lowercase letters indicate significant differences at the $p < 0.05$ level. BG, β-D-glucosidase; LAP, L-leucine aminopeptidase; NAG, β-1,4-*N*-acetylglucosaminidase; NP, neutral phosphatase.

Table S2. Effect of nanobubble watering with zinc oxide nanoparticles on activities of antioxidant enzymes in wheat roots (n = 3).

		APX Activity (nkat g⁻¹ FW)			CAT Activity (nkat g⁻¹ FW)			cwPOX Activity (nkat g⁻¹ FW)			DHAR Activity (nkat g⁻¹ FW)		
		**mean**	**SE**	**sig**	**mean**	**SE**	**sig**	**mean**	**SE**	**sig**	**mean**	**SE**	**sig**
ZnO0	W	0.229	0.008	b	0.124	0.021	a	0.127	0.000	a	2.348	0.013	a
	nW	0.286	0.016	a	0.115	0.005	a	0.127	0.000	a	2.034	0.127	b
ZnO500	W	0.121	0.013	c	0.159	0.014	a	0.127	0.000	a	2.125	0.070	ab
	nW	0.052	0.019	d	0.148	0.013	a	0.127	0.000	a	1.585	0.079	c
ZnO			**			*			n.s.			**	
nW			n.s.			n.s.			n.s.			**	
ZnO×nW			**			n.s.			n.s.			n.s.	

		GR Activity (nkat g⁻¹ FW)			GST Activity (nkat g⁻¹ FW)			MDHAR Activity (nkat g⁻¹ FW)			POX Activity (nkat g⁻¹ FW)		
		**mean**	**SE**	**sig**	**mean**	**SE**	**sig**	**mean**	**SE**	**sig**	**mean**	**SE**	**sig**
ZnO0	W	0.543	0.019	a	5.911	0.053	a	0.575	0.054	a	0.127	0.000	a
	nW	0.067	0.008	c	5.741	0.026	a	0.579	0.042	a	0.127	0.000	a
ZnO500	W	0.584	0.039	a	3.269	0.033	b	0.646	0.075	a	0.127	0.000	a
	nW	0.335	0.046	b	2.831	0.080	c	0.604	0.053	a	0.127	0.001	a
ZnO			**			**			n.s.			n.s.	
nW			**			**			n.s.			n.s.	
ZnO×nW			**			*			n.s.			n.s.	

		SOD Activity (nkat g⁻¹ FW)		
		**mean**	**SE**	**sig**
ZnO0	W	54.661	0.225	a
	nW	54.840	0.151	a
ZnO500	W	54.803	0.073	a
	nW	55.141	0.277	a
ZnO			n.s.	
nW			n.s.	
ZnO×nW			n.s.	

ZnO_{500} and ZnO_0 indicate irrigation with/without 500 mg L^{-1} ZnO solution, while W and nW indicate normal water and nanobubble-containing water. n.s., no significant difference; ** $p < 0.01$; * $p < 0.05$. Different small letters indicate significant difference at $p < 0.05$ level. APX, ascorbate peroxidase; CAT, catalase; cwPOX, cell wall peroxidase; DHAR, dehydroascorbate reductase; GR, glutathione reductase; GST, glutathione S-transferase; MDHAR, monodehydroascorbate reductase; POX, peroxidase; SOD, superoxide dismutase.

Table S3. Effect of nanobubble watering with zinc oxide nanoparticles on activities of antioxidant enzymes in wheat leaves (*n* = 3).

		APX Activity (nkat g^{-1} FW)			CAT Activity (nkat g^{-1} FW)			cwPOX Activity (nkat g^{-1} FW)			DHAR Activity (nkat g^{-1} FW)		
		**mean**	**SE**	**sig**	**mean**	**SE**	**sig**	**mean**	**SE**	**sig**	**mean**	**SE**	**sig**
ZnO0	W	2.089	0.033	ab	0.199	0.002	a	0.127	0.000	a	2.526	0.088	a
	nW	1.711	0.216	b	0.183	0.021	a	0.148	0.021	a	1.986	0.114	b
ZnO500	W	2.161	0.056	a	0.162	0.010	a	0.169	0.011	a	2.454	0.057	a
	nW	2.284	0.069	a	0.186	0.008	a	0.139	0.012	a	1.923	0.045	b
ZnO			*			n.s.			n.s.			n.s.	
nW			n.s.			n.s.			n.s.			**	
ZnO×nW			n.s.			n.s.			n.s.			n.s.	

		GR Activity (nkat g^{-1} FW)			GST Activity (nkat g^{-1} FW)			MDHAR Activity (nkat g^{-1} FW)			POX Activity (nkat g^{-1} FW)		
		**mean**	**SE**	**sig**	**mean**	**SE**	**sig**	**mean**	**SE**	**sig**	**mean**	**SE**	**sig**
ZnO0	W	1.260	0.037	d	4.901	0.097	b	0.752	0.031	b	0.127	0.000	a
	nW	2.611	0.051	a	5.934	0.029	a	0.641	0.027	b	0.127	0.000	ab
ZnO500	W	1.655	0.041	c	4.574	0.040	c	1.083	0.063	a	0.127	0.000	ab
	nW	2.264	0.011	b	4.724	0.062	bc	0.176	0.028	c	0.126	0.000	b
ZnO			n.s.			**			n.s.			n.s.	
nW			**			**			**			n.s.	
ZnO×nW			**			**			**			n.s.	

		SOD Activity (nkat g^{-1} FW)		
		**mean**	**SE**	**sig**
ZnO0	W	140.141	0.602	b
	nW	140.049	0.396	b
ZnO500	W	143.603	0.877	a
	nW	141.738	0.424	ab
ZnO			*	
nW			n.s.	
ZnO×nW			n.s.	

ZnO_{500} and ZnO_{0} indicate irrigation with/without 500 mg L^{-1} ZnO solution, while W and nW indicate normal water and nanobubble-containing water. n.s., no significant difference; ** $p < 0.01$; * $p < 0.05$. Different lowercase letters indicate significant differences at the $p < 0.05$ level. APX, ascorbate peroxidase; CAT, catalase; cwPOX, cell wall peroxidase; DHAR, dehydroascorbate reductase; GR, glutathione reductase; GST, glutathione S-transferase; MDHAR, monodehydroascorbate reductase; POX, peroxidase; SOD, superoxide dismutase.
